# Increased Virulence of Outer Membrane Porin Mutants of *Mycobacterium abscessus*

**DOI:** 10.3389/fmicb.2021.706207

**Published:** 2021-07-14

**Authors:** Vinicius C. N. de Moura, Deepshikha Verma, Isobel Everall, Karen P. Brown, Juan M. Belardinelli, Crystal Shanley, Megan Stapleton, Julian Parkhill, R. Andres Floto, Diane J. Ordway, Mary Jackson

**Affiliations:** ^1^Mycobacteria Research Laboratories, Department of Microbiology, Immunology and Pathology, Colorado State University, Fort Collins, CO, United States; ^2^Molecular Immunity Unit, Medical Research Council (MRC)-Laboratory of Molecular Biology, University of Cambridge Department of Medicine, Cambridge, United Kingdom; ^3^Wellcome Trust Sanger Institute, Hinxton, United Kingdom; ^4^Cambridge Centre for Lung Infection, Papworth Hospital, Cambridge, United Kingdom; ^5^Department of Veterinary Medicine, University of Cambridge, Cambridge, United Kingdom

**Keywords:** *Mycobacterium*, *abscessus*, porin, virulence, antibiotic resistance

## Abstract

Chronic pulmonary infections caused by non-tuberculous mycobacteria of the *Mycobacterium abscessus* complex (MABSC) are emerging as a global health problem and pose a threat to susceptible individuals with structural lung disease such as cystic fibrosis. The molecular mechanisms underlying the pathogenicity and intrinsic resistance of MABSC to antibiotics remain largely unknown. The involvement of Msp-type porins in the virulence and biocide resistance of some rapidly growing non-tuberculous mycobacteria and the finding of deletions and rearrangements in the porin genes of serially collected MABSC isolates from cystic fibrosis patients prompted us to investigate the contribution of these major surface proteins to MABSC infection. Inactivation by allelic replacement of the each of the two Msp-type porin genes of *M. abscessus subsp. massiliense* CIP108297, *mmpA* and *mmpB*, led to a marked increase in the virulence and pathogenicity of both mutants in murine macrophages and infected mice. Neither of the mutants were found to be significantly more resistant to antibiotics. These results suggest that adaptation to the host environment rather than antibiotic pressure is the key driver of the emergence of porin mutants during infection.

## Introduction

Two groups of organisms account for 70–95% of the pulmonary non-tuberculous mycobacteria (NTM) infections worldwide: The *Mycobacterium avium* complex (MAC) and the *Mycobacterium abscessus* complex (MABSC) (Park and Olivier, 2015). While MAC is still, overall, the most common cause of NTM infections in the United States (accounting for over 70% of NTM pulmonary infections), over the last 10 years, rapidly growing NTM of the *M. abscessus* complex (MABSC) [including *M. abscessus subsp. abscessus* (*Mabs*), *M. abscessus subsp. massiliense* [*Mmas*] and *M. abscessus* subsp. *bolletii*] have emerged as important human pathogens globally, causing an increasing number of pulmonary infections among patients with structural lung disease such as chronic obstructive pulmonary disease, bronchiectasis and cystic fibrosis (CF) (Floto and Haworth, 2015; Park and Olivier, 2015; Martiniano et al., 2019; Johansen et al., 2020). Detailed epidemiology and population-level whole genome sequencing studies have indicated that dominant circulating clones of *Mabs* and *Mmas* have recently emerged and are now present on every continent (Shang et al., 2011; Aitken et al., 2012; Bryant et al., 2013, 2016; Davidson et al., 2013; Tettelin et al., 2014). These clones are associated with worse clinical outcomes, have increased ability to infect and survive within macrophages, and cause more severe infections in mouse models (Shang et al., 2011; Bryant et al., 2016).

Despite recent advances in the genomics and genetics of MABSC organisms and the availability of a growing number of cellular and animal models of NTM infection (Medjahed and Reyrat, 2009; Ripoll et al., 2009; Bryant et al., 2016; Bernut et al., 2017), the molecular mechanisms underlying the intrinsic antibiotic resistance/tolerance, virulence and adaptation of MABSC species to becoming chronic pathogens of the lung are still poorly understood. Previous studies from our laboratory and others have established the important role played by outer membrane porins in the virulence and biocide resistance of rapidly growing mycobacteria including *M. smegmatis* and *M. chelonae* (Stephan et al., 2004; Sharbati-Tehrani et al., 2005; Danilchanka et al., 2008; Fabrino et al., 2009; Svetlikova et al., 2009; Purdy et al., 2009; Frenzel et al., 2011; Rodrigues et al., 2011). Msp-type porins represent the main general diffusion pathway for small hydrophilic molecules across the outer membrane of rapidly growing mycobacteria (Niederweis, 2003). Not only does their presence at the cell surface increase the permeability and susceptibility of *M. smegmatis* and *M. chelonae* to antibiotics and disinfectants (Stephan et al., 2004; Danilchanka et al., 2008; Svetlikova et al., 2009; Frenzel et al., 2011; Rodrigues et al., 2011), it was also found to impact the survival of *M. smegmatis* and that of an *M. tuberculosis mspA*-expressing strain inside phagocytic cells by increasing the susceptibility of the bacilli to killing by reactive nitrogen intermediates, ubiquitin-derived antimicrobial peptides and lysosomal enzymes (Sharbati-Tehrani et al., 2005; Fabrino et al., 2009; Purdy et al., 2009).

The reported involvement of porins in the virulence phenotype and biocide resistance of some rapidly growing mycobacteria, combined with the finding of deletions and rearrangements in the porin genes of serially collected *Mabs* and *Mmas* isolates from CF patients and genetic polymorphisms affecting the porin locus of MABSC isolates globally, led us to investigate the potential roles played by Msp-type porins in the adaptation of MABSC to the host and to antibiotic and disinfectant exposure.

## Materials and Methods

### Bacterial Strains and Culture Media

*Escherichia coli* DH5α, the strain used for cloning, was grown in LB Lennox (BD, Difco) medium at 37°C. *M. abscessus* subsp. *massiliense* (*Mmas*) CIP108297, a clinical isolate recovered from the sputum and bronchoalveolar fluid of a patient with hemoptoic pneumonia (Adekambi et al., 2004) was grown at 37°C in Middlebrook 7H9-OADC broth (BD, Difco) supplemented with 0.05% Tween 80, Mueller-Hinton II broth (BD, Difco), minimal Glycerol-Alanine-Salt (GAS) medium supplemented with 0.05% tyloxapol (pH 6.6), or on Middlebrook 7H11-OADC agar (BD, Difco). Kanamycin (Kan), hygromycin (Hyg) and streptomycin (Str) were added to final concentrations of 400, 1,000, and 200 μg/ml, respectively.

### Generation of Porin Knock-Out Mutants

Recombineering was used to inactivate the MMCCUG48898_0905 (herein renamed *mmpA*) and MMCCUG48898_0906 (herein renamed *mmpB*) genes of *Mmas* CIP108297 by allelic replacement. To this end, the Gp60 and Gp61 recombineering proteins from mycobacteriophage Che9c were expressed from the replicative plasmid pJV53-XylE under control of an acetamide-inducible promoter as described (van Kessel and Hatfull, 2007; van Kessel et al., 2008; Calado Nogueira de Moura et al., 2014). Acetamide-induced *Mmas* CIP108297 harboring pJV53-XylE were electro-transformed with linear allelic exchange substrates encompassing the *mmpA* and *mmpB* loci and double-crossover mutants were isolated on Str-containing medium. The linear allelic substrate used to delete the *mmpA* locus was generated by bracketing the streptomycin-resistance cassette from pHP45Ω with 571–500 bp of upstream and downstream DNA sequence flanking the internal *Xmn*I restriction site of *mmpA*, respectively. The linear allelic substrate used to delete the *mmpB* locus was generated by similarly bracketing the streptomycin-resistance cassette with 565–357 bp of upstream and downstream DNA sequence immediately flanking the internal *Xmn*I restriction site of *mmpB*. The linear allelic substrate used to try and delete both porin genes simultaneously was generated by bracketing the streptomycin-resistance cassette with 571 bp of DNA sequence located upstream the *Xmn*I site of *mmpA* and 357 bp of DNA sequence downstream the *Xmn*I site of *mmpB*.

pOMK-*mmpA*, pOMK-*mmpB*, and pOMK-*mmpAB*, the replicative plasmids used for the complementation of the knock-out mutants, were constructed by cloning *mmpA*, *mmpB* or a 2.1 kb region encompassing both genes in the multicopy plasmid pOMK (Calado Nogueira de Moura et al., 2014). The *mmpA* and *mmpB* genes are expressed from their own promoter in the complementation constructs. The sequences of the primers used to generate the different constructs are provided in Supplementary Table 1.

### Phenotypic Assays

The susceptibility of *Mmas* to commercial glutaraldehyde (GTA) in suspension tests, MIC determinations and [U-^14^C]-glucose (5 mCi/mmol; American Radiolabeled Chemicals) uptake experiments were performed as described in Calado Nogueira de Moura et al., 2014. For *in vitro* NO susceptibility studies, *Mmas* strains (approximately 2 × 10^7^ cells) grown in 7H9-OADC medium were resuspended in 1 ml phosphate buffer (pH 7.4) and exposed to 10 mM Spermine NONOate (Cayman Chemical) for 4–24 h at 25°C at which time point viable CFUs were enumerated by plating serial dilutions of the cell suspensions on 7H11-OADC agar.

### Immunoblot Analysis of Outer Membrane Porins

Porin extraction and immunoblot analysis using anti-MspA antibodies (Stahl et al., 2001) was carried out as described previously (Calado Nogueira de Moura et al., 2014).

### RNA Preparation, Reverse Transcription and qRT-PCR

*Mmas* RNA was extracted from 5-ml cultures grown to an OD_600_ of 0.2 using the Direct-zol RNA Miniprep kit (Zymo Research) per the manufacturer’s instructions. Reverse transcription reactions were carried out using the Superscript V kit (Invitrogen), and qRT-PCRs were run using the SsoFast Evagreen Supermix kit (Bio-Rad) as per the manufacturers’ protocols and analyzed on a CFX96 real-time PCR machine (Biorad). PCR conditions: 98°C (2 min; enzyme activation), followed by 35 cycles of 98°C (5 sec; denaturation) and 55°C (5 sec; annealing/extension). Mock reactions (no reverse transcription) were done on each RNA sample to rule out DNA contamination. The target cDNA was normalized internally to the *sigA* cDNA levels in the same sample. The primer sequences are provided in Supplementary Table 1.

### Genome Dataset, Mapping, *de novo* Assembly and Annotation

A previously published global collection of MABSC isolates, consisting of 526 isolates associated with pulmonary infections and 189 isolates collected from nine separate outbreak locations in Brazil, was used in this study (Bryant et al., 2016; Everall et al., 2017). These isolates were further supplemented by an additional 665 longitudinal isolates associated with 190 patients with Cystic Fibrosis (CF) (Bryant et al., 2021). The Illumina Hiseq 2,500 paired end raw reads of all the isolates were mapped using BWA-MEM v0.7.12 to the reference genome *Mabs* ATCC 19977 (Li. 2013). Assemblies for all the isolates were generated and optimized using Velvet and Velvet optimizer, respectively (Zerbino and Birney, 2008). The final draft assemblies were annotated using PROKKA (Seemann, 2014).

### Phylogenetic Analysis

An alignment was produced from the mapping of 526 isolates associated with pulmonary infections and 188 isolates associated with the post-surgical wound infection epidemic in Brazil to the *Mabs* ATCC 19977 reference genome, using SAMtools v1.2 and BCFtools v4.2, with parameters previously described (Li et al., 2009; Harris et al., 2010). Briefly, a variant was inferred if the following criteria were met: A mapping quality of 20, a base call quality of 50, eight reads supporting the alternative allele and three reads supporting the alternative allele on either strand. Heterozygous SNP sites were masked. An alignment of the variable positions was created using SNP-sites and used to produce a maximum likelihood phylogeny with RAxML (Stamatakis, 2014; Page et al., 2016). Hundred bootstrap replicates were performed.

### Detection of Msp-Like Porin Gene Homologs in MABSC Genomes

A local tblastx search was performed to detect homologs of *msp*-like porin genes in the MABSC genomes. A local database was made using the nucleotide sequences of *mmpA* (MAB_1080), *mmpB* (MAB_1081) and the possible *mmpC* homolog (MAB_2800). The assemblies of 1,382 MABSC isolates were queried against this database with *msp*-like porin gene homologs predicted if a match had a percent identity greater than 50%, *E*-value < 0.05 and a match length greater than or equal to 50% of the length of the reference gene of *mmpA* (672 bps), *mmpB* (669 bps) and *mmpC* (741 bps).

### Analysis of the mspA/mspB Porin Locus Configuration; Identification of the Fusion Position, and Identification of Loss of Function Mutations in *mmpA* and *mmpB*

The coverage over the porin locus containing the *mmpA* and *mmpB* genes was determined for each isolate using SAMtools v1.3, with a mapping quality threshold of 20 (probability mapped to correct location 99%), base call quality of 30 (base call is 99.9% accurate) (Li et al., 2009). Presence of both *msp*-like porin genes at this locus was determined by an average depth of coverage greater than eight in the 436 bp intergenic region between *mmpA* and *mmpB* and an average depth of coverage greater than eight over both *mmpA* and *mmpB*. Deletion of both *mmp*-like porin genes at this locus was determined by the absence of coverage (average of less than eight reads mapped) in the intergenic region between *mmpA* and *mmpB* and a reduction in depth of coverage greater than 20 between the 1,000 bp region upstream of the start of the porin loci and the 94 bp signal peptide region of *mmpA*. The presence of a single *mmp*-like porin gene at this locus was determined by absence of coverage in the intergenic region between *mmpA* and *mmpB* (average of less than eight reads mapped) and no reduction in depth of coverage greater than 20 between the 1,000 bp flanking region upstream of the porin loci and the 94 bp signal peptide region of *mmpA*. The figures were made using easyfig (Sullivan et al., 2011). A two tailed Fisher’s exact test was performed to determine if there was an association between the *mmpA* and *mmpB* porin locus configuration and isolates classified as clustered and more virulent and those classified as unclustered by Bryant et al. (2016).

Where only one *mmp*-like porin gene was present at the *mmpA/mmpB* porin locus, an alignment of the nucleotide sequence of this gene, and the reference *mmp*A (MAB_1080) and *mmpB* (MAB_1081) genes was created using muscle v3.8.31 (Edgar, 2004). The position at which the recombination event occurred was predicted based on where the *mmp*-like porin gene of the isolate with one *mmp*-like porin gene at the *mmpA/mmpB* porin locus changed from being more similar to *mmpA* to being more similar to *mmpB*.

The raw reads of the 1,382 isolates were further mapped to the *Mabs* ATCC 19977 *mmpA/mmpB* porin locus using BWA-MEM v0.7.12 (Li, 2013). Variants were called using SAMtools v1.2 and BCFtools v4.2 with the following parameters (Li et al., 2009): A base call quality of 30 and mapping quality of 20, a minimum of eight reads supporting the alternative base, with a minimum of three reads supporting the alternative base on each strand. Variants, including Indels, were only determined for isolates predicted to encode both *mmpA* and *mmpB* at this locus.

### Mice

Specific-pathogen-free female SCID and GMCSF knock-out mice, from 6 to 8 weeks old, were purchased from the Jackson Laboratories, Bar Harbor, Maine. Mice were maintained in the Biosafety Level III animal laboratory at Colorado State University, and were given sterile water, mouse chow, bedding, and enrichment for the duration of the experiments. The specific pathogen-free nature of the mouse colonies was demonstrated by testing sentinel animals. All experimental protocols were approved by the Animal Care and Usage Committee of Colorado State University. The CSU animal assurance welfare number is A3572-01.

### Bone Marrow-Derived Macrophage Infections

Bone marrow-derived macrophages (BMDM) from GMCSF and SCID knock-out mice were infected with mycobacteria at a one bacterium per one macrophage ratio and, 2 h later, the monolayers were washed to remove extracellular bacilli. The numbers of intracellular mycobacteria were measured by plating. Briefly, monolayers were washed at each time point to remove extracellular bacilli and 1 ml double-distilled H_2_O containing 0.05% Tween 80 was added to monolayers and incubated for 10 min to lyse macrophages. After passing through a 26-gauge needle five times, the lysates were serially diluted and plated onto 7H11-OADC agar. Monolayers that weren’t lysed were replenished with fresh medium as previously described (Shang et al., 2011; Obregon-Henao et al., 2015). BMDM viability was assayed by trypan blue exclusion and determined by flow cytometry using BD Viability Counting Beads, as described by the manufacturer (BD PharMingen, San Jose, CA United States) cell viability-staining methods.

### SCID Mice Infection

SCID mice were challenged with WT, mutant and complemented mutant strains using an infection calibrated to deliver 1.0 × 10^6^ bacilli per animal. At days 1 and 15 following infection, bacterial loads in the lungs, spleen and liver, and lung histology were determined. Bacterial counts were determined by plating serial dilutions of organ homogenates on 7H11-OADC agar and counting colony-forming units after 5–10 days of incubation at 30°C. A total of five animals were infected for each time point.

### Histological Analysis

The whole lung from each mouse was fixed with 10% formalin in phosphate buffered saline (PBS). Tissue sections were stained using hematoxylin and eosin and acid-fast stain as previously reported (Bryant et al., 2016).

### Statistical Analysis

Data are presented using the mean values from five mice per group and from values from replicate samples and triplicate macrophage infection assays. The unpaired Student’s *t*-test test was used to assess statistical significance between infection groups.

## Results

### Msp-Like Porin Genes in the Genomes of Reference MABSC Isolates

The reference strains *Mmas* CIP108297 (alias CCUG 48898) and *Mabs* ATCC 19977 harbor two clustered Msp-like porin genes annotated as *MMCCUG48898_0905*/*MMCCUG48898_0906* and *MAB_1080*/*MAB_1081*, respectively (Ripoll et al., 2009; Tettelin et al., 2012). We renamed the two *Mmas* genes *mmpA* and *mmpB* for *Mycobacterium abscessus* subsp. *massiliense* porins A and B. In addition, the genomes of *Mmas* CIP108297 and *Mabs* ATCC 19977 encode a third, more distantly related, porin gene (MAB_2800 in *Mabs* ATCC 19977) which we renamed *mmpC* and six other proteins sharing 22–53% identity with MmpC annotated as putative porins (MAB_2799; MAB_1321; MAB_3154c; MAB_2329c; MAB_0734 and MAB_1891). The mature MmpA and MmpB porin products share 97% identity between them and 72–73% identity with MspA from *M. smegmatis* (Supplementary Figure 1) (98.9–100% identity with the *Mabs* ATCC 19977 porin products, MAB_1080 and MAB_1081). MmpC shares only 22% identity (27.8% similarity) with MmpA and MmpB from *Mabs* ATCC 19977 and 22.3% identity (28.4% similarity) with MspA from *M. smegmatis* mc^2^155. 463 bp of nucleotide sequence separate *mmpA* from *mmpB* in *Mmas* CIP108297 and *Mabs* ATCC 19977 suggesting that the two genes are probably each monocistronic.

### Variation in Genome Organization at the Porin Locus of MABSC Clinical Isolates

At least one (*mmpA/mmpB*- or *mmpC*-like) porin gene homolog was detected in all the MABSC genomes used in this study (*n* = 1,382). However, analysis of the depth of coverage of the mapped reads over the *mmpA* and *mmpB* porin loci of the 1,382 MABSC genomes used herein showed that there was variation in the number of porin genes encoded at this position. Overall, 1,059 isolates encoded both *mmpA* and *mmpB* porin genes at this locus, 312 isolates encoded a single *mmp*-like porin gene (a fusion of *mmpA* and *mmpB*; see further) and 11 isolates encoded no *mmp-*like porin genes at this locus.

**FIGURE 1:**
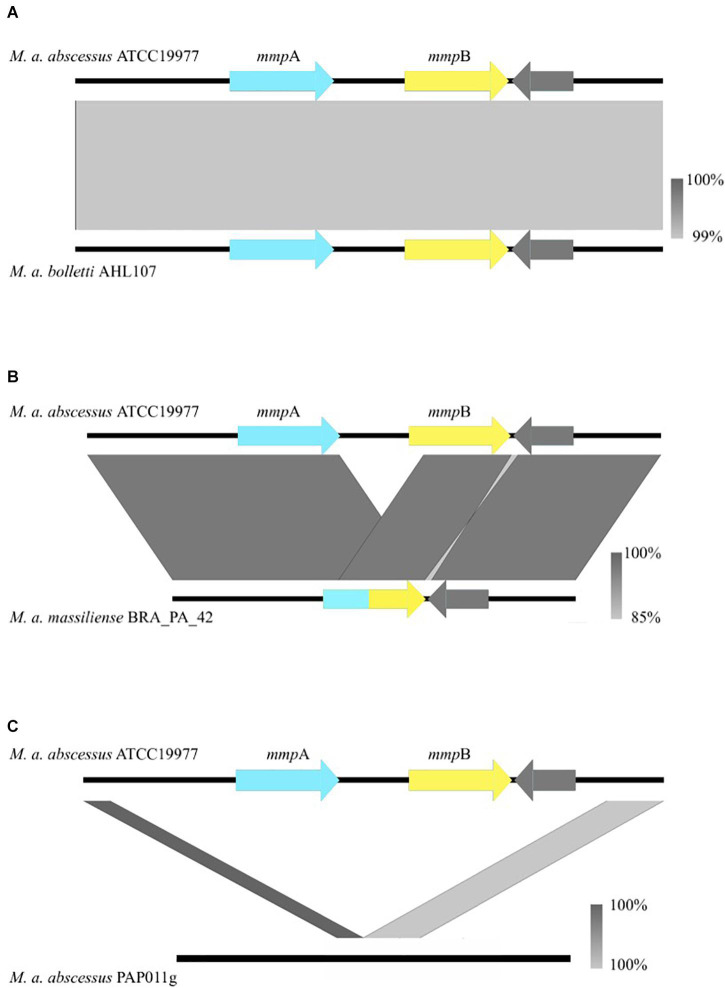
Nucleotide BLAST comparisons between the *mmpA/mmpB* porin locus of *Mabs* ATCC 19977 and isolates that encode the variants observed in the porin locus both across the global population and longitudinally. The gray bars illustrate the various rearrangements [or no rearrangement in the case of **(A)]** observed in clinical isolates and are color-coded to indicate the percentage identity of the genomic sequences of the two isolates that are being compared. **(A)** Is an example of when *mmpA* and *mmpB* are both present. **(B)** Is an example of the fusion of *mmpA* and *mmpB*, resulting in a single *mmp*-like gene. **(C)** Is an example of the complete deletion of both *mmp*-like porin genes at the porin locus. Porin genes are colored in blue and yellow.

Of the 526 isolates associated with pulmonary infections, 469 isolates encoded two *mmp*-like porin genes at this locus (Figure 1A), 54 encoded a single *mmp-*like porin fusion gene (Figure 1B) and three isolates encoded no *mmp-*like porin genes at this locus (Figure 1C). A single *mmp*-like porin gene, a fusion of *mmpA* and *mmpB*, was observed in 180 of 189 isolates associated with the post-surgical wound infection epidemic in Brazil (Duarte et al., 2009; Leao et al., 2010; Everall et al., 2017), while the remaining 9 isolates encoded two *mmp*-like porin genes at this locus.

Short indels, leading to the loss of function of the protein, were observed in *mmpA* in seven isolates associated with pulmonary infections. One isolate incurred a non-synonymous mutation resulting in a premature stop codon, W220^∗^.

Changes in genome organization at the *mmpA/mmpB* locus were also observed in isolates collected from the same patient over time. Isolates from 15 of the 190 CF patients for which longitudinal samples were available were found to vary in the number of *mmp*-like porin genes encoded by this locus. Isolates from 12 of the 15 patients either encoded one or two *mmp*-like porins, one patient’s isolates encoded either two *mmp*-like porin genes or no *mmp*-like porin genes, one patient’s isolates encoded either one *mmp*-like porin gene or no *mmp*-like porin genes and the final patient’s isolates varied between encoding either two, one or no *mmp*-like porin genes at the *mmpA/mmpB* locus. A further four patients’ isolates were shown to have accumulated indels in *mmpA* over time. No non-synonymous mutations were found to have occurred within patients over time.

**FIGURE 2:**
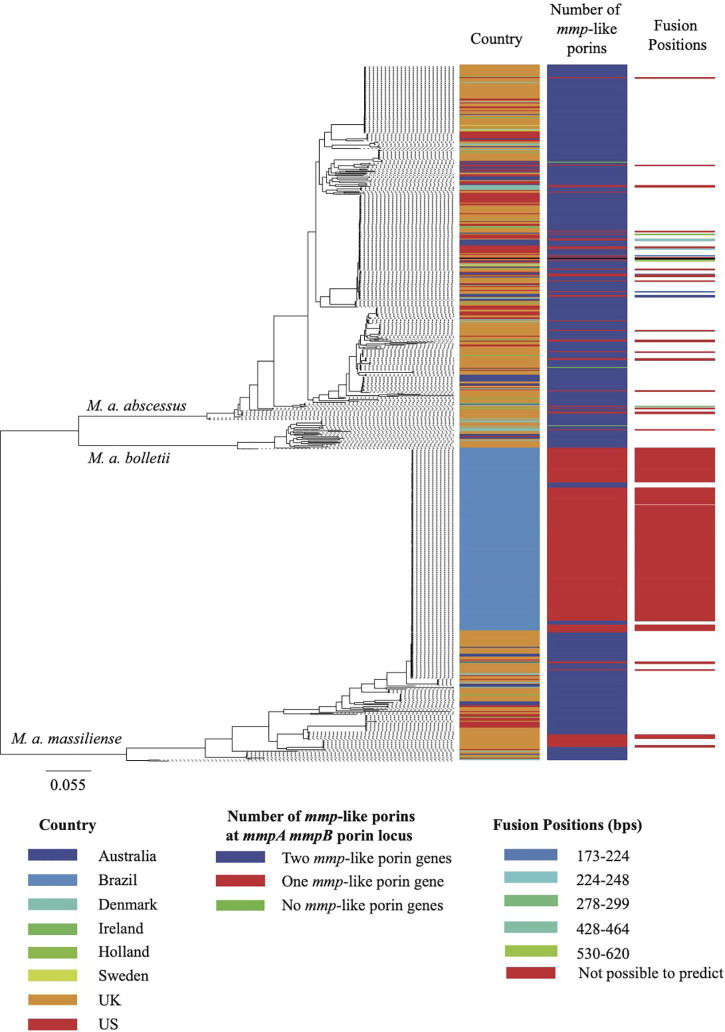
Maximum likelihood phylogeny of the MABSC global population. The global population shown here consist of 526 isolates associated with pulmonary infections and 188 isolates associated with the epidemic of post-surgical wound infections in Brazil. The metadata aligned shows, for each isolate, the country of isolation, the predicted composition of the porin locus and the isolates for which the fusion position was predicted. The scale bar on the phylogeny represents the number of substitutions per variable site.

As noted above, where isolates were found to have only one *mmp*-like porin gene at the *mmpA*/*mmpB* porin locus, a recombination event had occurred leading to the fusion of *mmpA* and *mmpB*. We examined the sequences of the 312 predicted *mmpA/mmpB* fusion genes to determine where in the sequence the fusion occurred. Of the 312 predicted *mmpA/mmpB* fusion genes, it was only possible to extract the complete sequence of the gene from the draft assembly of 297 isolates. It was possible to predict the fusion position for 38 of these isolates, with six unique fusion positions predicted which spanned close to the entire length of *mmpA* (Figure 2). All six fusion positions were also seen in isolates where changes in the number of *mmp*-like porin genes encoded at the *mmpA/mmpB* porin locus were observed over time within a patient. All the predicted fusion positions fall after the signal peptide sequence at the N-terminal of MmpA and, therefore, all the fusion genes use the signal peptide sequence of this porin.

### Generation of mmpA and mmpB Knock-Out Mutants of Mmas

Because the changes occurring at *mmpA*/*mmpB* loci of longitudinally isolated strains from CF patients was suggestive of the involvement of MmpA and MmpB in the adaptation of MABSC to the host, subsequent studies were undertaken to determine whether these naturally occurring mutations arose as a result of the selective pressure of antibiotic treatment or as part of a more general adaptation of the bacterium to stresses imposed by the host environment.

**FIGURE 3:**
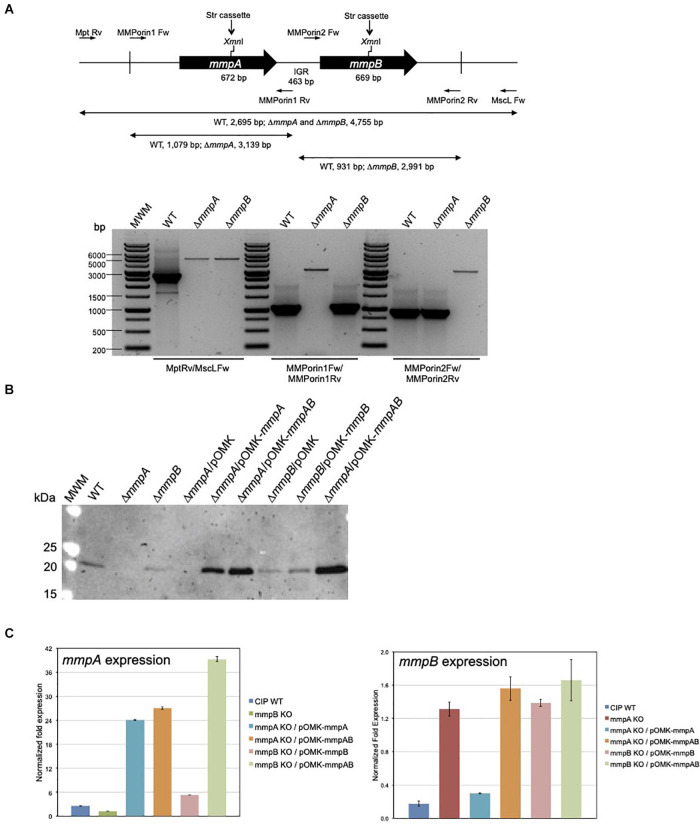
Gene replacement at the *mmpA* and *mmpB* porin loci of *Mmas*. **(A)** Porin gene cluster of *Mmas* CIP108297. The positions of the primers used to generate the allelic exchange substrates and analyze the candidate mutants are indicated along with the expected sizes of the PCR fragments. Candidate *mmpA* and *mmpB* mutants were analyzed by PCR using different sets of primers, including Mpt-Rv/MscL-Fw located outside the allelic exchange substrates, MMPorin1 Fw/MMPorin1 Rv, and MMPorin2 Fw/MMPorin2 Rv (see Supplementary Table 1). MWM, molecular weight marker; IGR, intergenic region. **(B)** Immunoblot analysis of porin production in the WT, mutant and complemented mutant strains. Strains were grown in 7H9-OADC-Tween 80 broth at 30°C to mid-log phase (OD600 = 1) and porins were selectively extracted from whole cells at 100°C using 0.5% *n*-octylpolyoxyethylene as a detergent as described (Heinz et al., 2003). Protein samples prepared from the same amount of cells for each strain were denatured by boiling in 80% DMSO followed by acetone precipitation (Heinz et al., 2003). Denatured proteins were loaded volume to volume, separated by SDS-PAGE, blotted onto a nitrocellulose membrane, and porins were detected using rabbit antiserum to purified MspA (Stahl et al., 2001). Immune complexes were detected by chemiluminescence (Pierce, ELC) and semi-quantified using the Image Lab software (Biorad). The expected size of the mature MmpA and MmpB porins is 19.65 kDa. **(C)** *mmpA* and *mmpB* mRNA levels in the WT, mutant and pOMK complemented mutant strains. The target cDNA was normalized internally to the *sigA* cDNA in the same sample. Ratios of *mmpA*/*sigA* or *mmpB*/*sigA* mRNA are means ± standard deviations (*n* = 3 qRT-PCR reactions). RNA extractions and qRT-PCR experiments were performed twice using independent culture batches and the results of one representative experiment are shown.

To investigate the role of MmpA and MmpB in the physiology and virulence of MABSC, we first sought to knock-out each of these two porin genes of *Mmas* CIP108297 by allelic replacement. To this end, the recombineering method was used wherein *Mmas* CIP108297 expressing the Gp60 and Gp61 recombineering proteins from mycobacteriophage Che9c from pJV53-XylE (van Kessel and Hatfull, 2007; van Kessel et al., 2008; Calado Nogueira de Moura et al., 2014) was electrotransformed with linear allelic exchange substrates designed to replace the entire *mmpA* or *mmpB* loci with a streptomycin-resistance cassette. Twelve to 55 double crossover candidates were selected for each gene and analyzed by PCR. PCR analysis confirmed that allelic replacement had occurred in 18% of the *mmpA* candidate mutants and 50% of the *mmpB* candidate mutants (Figure 3A). Two passages in liquid broth devoid of Kan followed by plating on Str-containing agar yielded single *mmpA* and *mmpB* mutant clones devoid of pJV53-XylE plasmid. In contrast, three independent attempts to inactivate both porin genes simultaneously yielded no double crossover mutant suggesting that, similar to the situation in *M. smegmatis* (Stephan et al., 2005), the presence of at least one functional porin gene is required for *Mmas* CIP108297 growth under the experimental conditions used herein. Given that our genomic analysis identified 11 isolates devoid of *mmpA* and *mmpB* genes, one may only speculate that the *mmpC* gene and homologs present in these isolates can compensate for the lack of *mmpA* and *mmpB* in a way that is not achievable in *Mmas* CIP108297.

### Impact of mmpA and mmpB Deficiency on Porin Production and Activity

Porin production in the mutant strains was analyzed by Western blot using polyclonal antibodies to the MspA porin of *M. smegmatis* (Stahl et al., 2001). While porin products of the expected size (19.6 kDa) were detected in the wild-type (WT) *Mmas* CIP108297 strain, they were barely detectable in the *mmpA* knock-out; the intensity of the porin signal in this mutant was only about 6–10% of that detected in the WT parent. In comparison, the intensity of the band was decreased by less than 40–50% in the *mmpB* knock-out (Figure 3B). Complementation of the mutants with their respective porin gene or with both porin genes expressed from the replicative multicopy plasmid pOMK increased porin production in the *mmpA* knock-out relative to the WT parent 3.4- and 5-fold, respectively, and that in the *mmpB* knock-out mutant 1- and 10-fold, respectively.

Porin expression in the two mutants was further analyzed by qRT-PCR to check for a potential polar effect of the inactivation of *mmpA* on the expression of *mmpB* and for the existence of compensatory mechanisms regulating the level of expression of the two porin genes. The result of two independent experiments indicated that while the level of expression of *mmpA* was only about 1.3–2-fold less in the *mmpB* mutant relative to the WT strain, the expression of *mmpB* increased 4–7-fold in the *mmpA* knock-out (Figure 3C). qRT-PCR analysis further revealed that the level of expression of *mmpA* relative to *sigA* was > 14-fold greater than that of *mmpB* in WT *Mmas* CIP108297, thereby confirming MmpA as the major porin product of this isolate and explaining the more dramatic effect of knocking-out *mmpA* over *mmpB* on the total porin content of this strain (Figure 3B).

**FIGURE 4:**
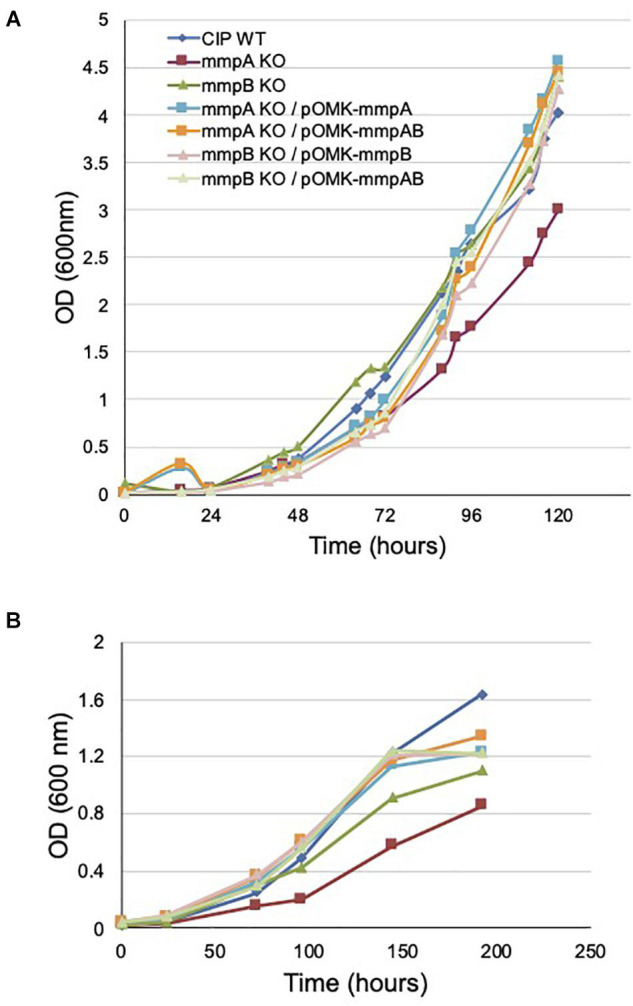
Growth rates of wild-type *Mmas*, its isogenic *mmpA* and *mmpB* knock-out mutants and the complemented porin mutants in GAS-tyloxapol **(A)** and glucose-based medium **(B)** at 37°C. Shown are representative results of two independent experiments. The glucose-based minimal medium consists of 20 mM asparagine, 1.5 mM KH_2_PO_4_, 0.75 mM Na_2_HPO4, 50 μM FeCl_3_, 1.6 mM MgSO_4_, 4.5 micromolar CaCl_2_, 3.5 μM ZnSO_4_, 0.59 micromolar MnSO_4_, 10 mM glucose and 0.05% tyloxapol in 50 mM MOPS (pH 7.0).

As expected, the disruption of *mmpA* impacted more dramatically the growth rate of *Mmas* CIP108297 than that of *mmpB*, the latter mutant growing at a similar rate as its WT parent in minimal GAS (Glycerol-Alanine-Salts) medium (Figure 4A) and at an intermediate rate between the *mmpA* knock-out and the WT strain in glucose-based medium (Figure 4B). Complementation with the individual or combined *mmpA* and *mmpB* genes restored WT growth in the mutants (Figures 4A,B).

**FIGURE 5:**
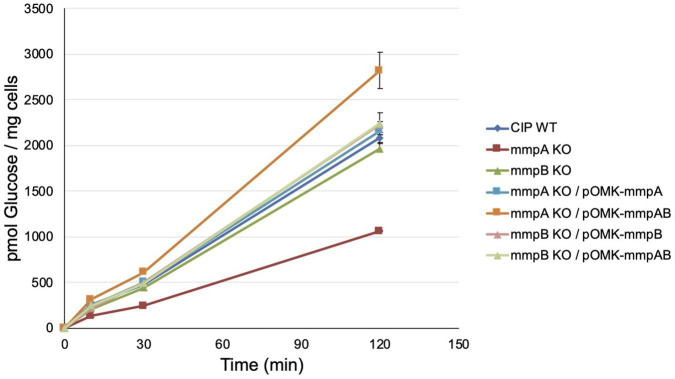
Glucose uptake by wild-type *Mmas*, the *mmpA* and *mmpB* knock-out mutants and the complemented porin mutant strains. The accumulation of [U-^14^C]glucose by the strains over time was measured as described (Calado Nogueira de Moura et al., 2014). Glucose uptake was measured in duplicate samples and the average values are shown with their standard deviations. Glucose uptake rates were calculated on the first 120 min of the reactions.

Consistent with these observations, the *mmpA* knock-out mutant presented a significantly reduced [^14^C]-glucose uptake rate relative to the WT parent (8.75 ± 0.03 vs. 17.11 ± 0.47 pmoles of glucose/mg cells/min) which was restored to WT levels upon complementation with *mmpA* (17.86 ± 0.41 pmoles of glucose/mg cells/min), and beyond WT levels upon complementation with *mmpA* and *mmpB* (23.35 ± 1.67 pmoles of glucose/mg cells/min) (Figure 5). The *mmpB* knock-out mutant, in contrast, was as proficient at taking up [^14^C]-glucose as WT *Mmas* CIP108297 in this assay (16.23 ± 0.58 and 17.11 ± 0.47 pmoles of glucose/mg cells/min, respectively).

### Susceptibility of the Mmas Porin Knock-Out Mutants to Glutaraldehyde, Antibiotics, and Nitrosative Stress

Because of the reported impact of porins on the susceptibility of *M. smegmatis* and *M. chelonae* to biocides and nitrosative stress (Fabrino et al., 2009; Svetlikova et al., 2009; Frenzel et al., 2011), we next set out to test the resistance of the *mmpA* and *mmpB* knock-out mutants to a number of stresses including various classes of antibiotics used in the clinical treatment of fast-growing mycobacterial infections, the high-level disinfectant glutaraldehyde (GTA), and nitric oxide (NO). Apart from a modest 2–4-fold increase in the resistance of the *mmpA* knock-out mutant to ciprofloxacin and imipenem, the two mutants presented susceptibility patterns to antibiotics (Table 1) and GTA (Supplementary Figure 2) comparable to those of their WT parent. In particular, both mutants displayed WT susceptibilities to drugs used in the clinical treatment of MABSC infections such as clarithromycin, erythromycin, azithromycin, amikacin, cefoxitin, linezolid and tigecycline. The *mmpA* and *mmpB* knock-outs further displayed WT susceptibility to NO *in vitro* (Supplementary Figure 3).

**TABLE 1:** Susceptibility of the *Mmas* porin mutants to antibiotics.

Strains	AMI	APR	AZI	CIP	CLA	ERY	TET	KAN	CEF	IMI	TIG	LIN
*Mmas* CIP108297	16–32	2	1–2	32–64	0.25	8–16	32	8	64	32	2–4	16
*Mmas*Δ*mmpA*	32	2–4	1–2	128	0.5	8	8	8	64	64–128	4	16
*Mmas*Δ*mmpB*	16–64	4	2	32–64	0.5	16	16	8	64	32	4	16
*Mmas*Δ*mmpA*/pOMK-*mmpA*	64	2	2	32	1	32	8	nd	64	64	4	16
*Mmas*Δ*mmpA*/pOMK-*mmpAB*	32	4	4	32	1	16	16	nd	64	64	4	16
*Mmas*Δ*mmpB*/pOMK-*mmpB*	32	4	2	32	0.25	8	8	nd	64	32	2–4	16
*Mmas*Δ*mmpB*/pOMK-*mmpAB*	32	4	2	32	0.5	8	8	nd	64	32	4	16

*MIC were determined in Mueller-Hinton II broth and MIC values are in μg/ml.AMI, amikacin; APR, apramycin; AZI, azithromycin; CEF, cefoxitin; CIP, ciprofloxacin; CLA, clarithromycin; ERY, erythromycin; IMI, imipenem; KAN, kanamycin; LIN, linezolid; TET, tetracycline; TIG, tigecycline. MIC determinations were performed two to three times on independent culture batches. nd, not determined.*

### Growth of Mmas Porin Mutants in SCID and GMCSF Knock-Out Mouse Bone Marrow-Derived Macrophages (BMDM)

**FIGURE 6:**
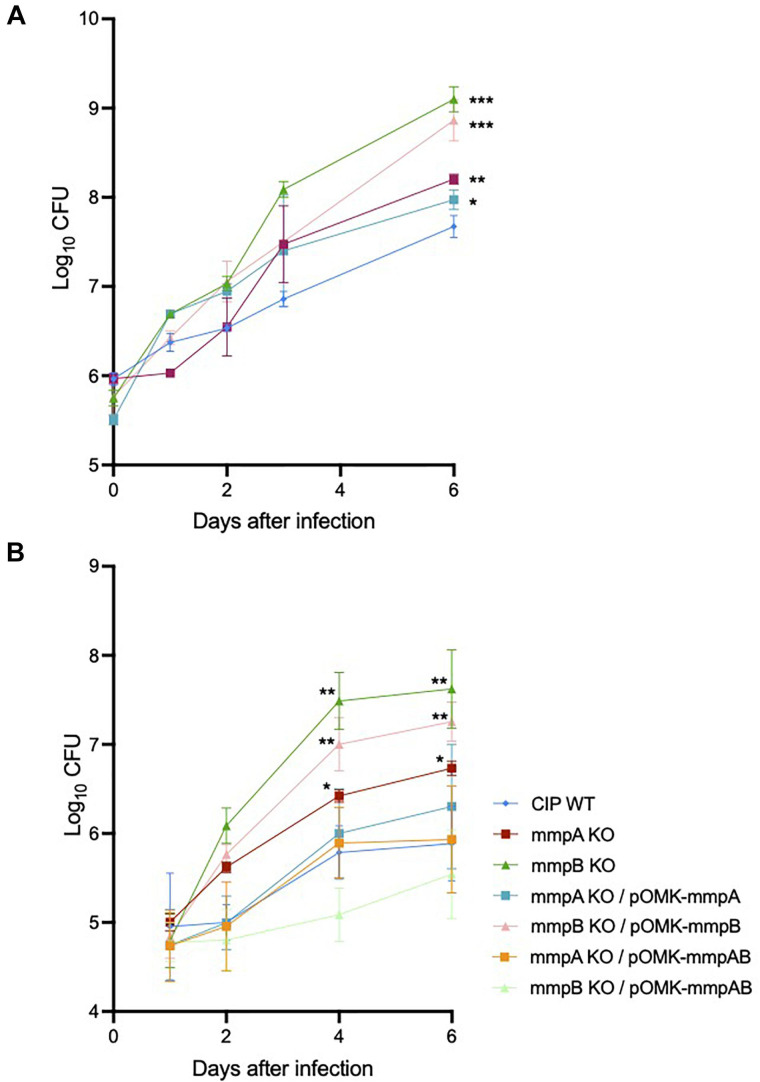
Increased intracellular replication of the porin mutants in SCID and GMCSF knock-out mouse bone marrow-derived macrophages. **(A)** Intracellular growth of the WT and mutant strains in SCID BMDM. BMDM were infected with *Mmas* strains at a MOI of 1:1. Viable intracellular bacteria were determined by CFU counting after 2 h of infection or at 1, 2, 3 and 6 days after infection. Values shown are the mean ± SEM from three infected macrophage monolayers. **(B)** Intracellular growth of the WT, mutant and complemented mutant strains in GMCSF BMDM. BMDM were infected with *Mmas* strains at a MOI of 1:1. Viable intracellular bacteria were determined by CFU counting after 2 h of infection or at 1, 2, 4, and 6 days after infection. Values shown are the mean ± SEM from three infected macrophage monolayers. Asterisks denote statistical differences between the growth of the knock-out mutants (or the *mmpB* KO complemented with *mmpB* only) and the WT parent strain pursuant to the Student’s *t*-test (*P < 0.05; **P < 0.01; ***P < 0.005).

We investigated the capacity of *Mmas* reference strain CIP108297 and corresponding porin knock-out mutants to replicate and persist in BMDM to evaluate their virulence. The growth of each strain was determined in BMDM prepared from SCID and GMCSF knock-out mice by the method of plating to measure colony-forming units (CFU) over a period of 6 days. While no significant difference in bacterial uptake was noted between strains, both mutants demonstrated significantly increased replication in SCID (Figure 6A) and GMCSF knock-out (Figure 6B) BMDM compared to their WT parent, with the *mmpB* mutant consistently displaying the most acute virulence phenotype of the two. Throughout the experiment, BMDM cellular viability (as assessed by the percentage of propidium iodide positive dead cells) was above 95% and similar for all strains indicating that the porin mutants were not more cytotoxic than their WT parent in these macrophage models (data not shown). The replication of the mutants in macrophages was restored to or below WT levels upon genetic complementation with both porin genes (Figure 6B), and between WT and knock-out levels upon complementation with the individual WT copy of *mmpA* or *mmpB* (Figures 6A,B).

### Pathogenicity of the Porin Knock-Out Mutants in SCID Mice

**FIGURE 7:**
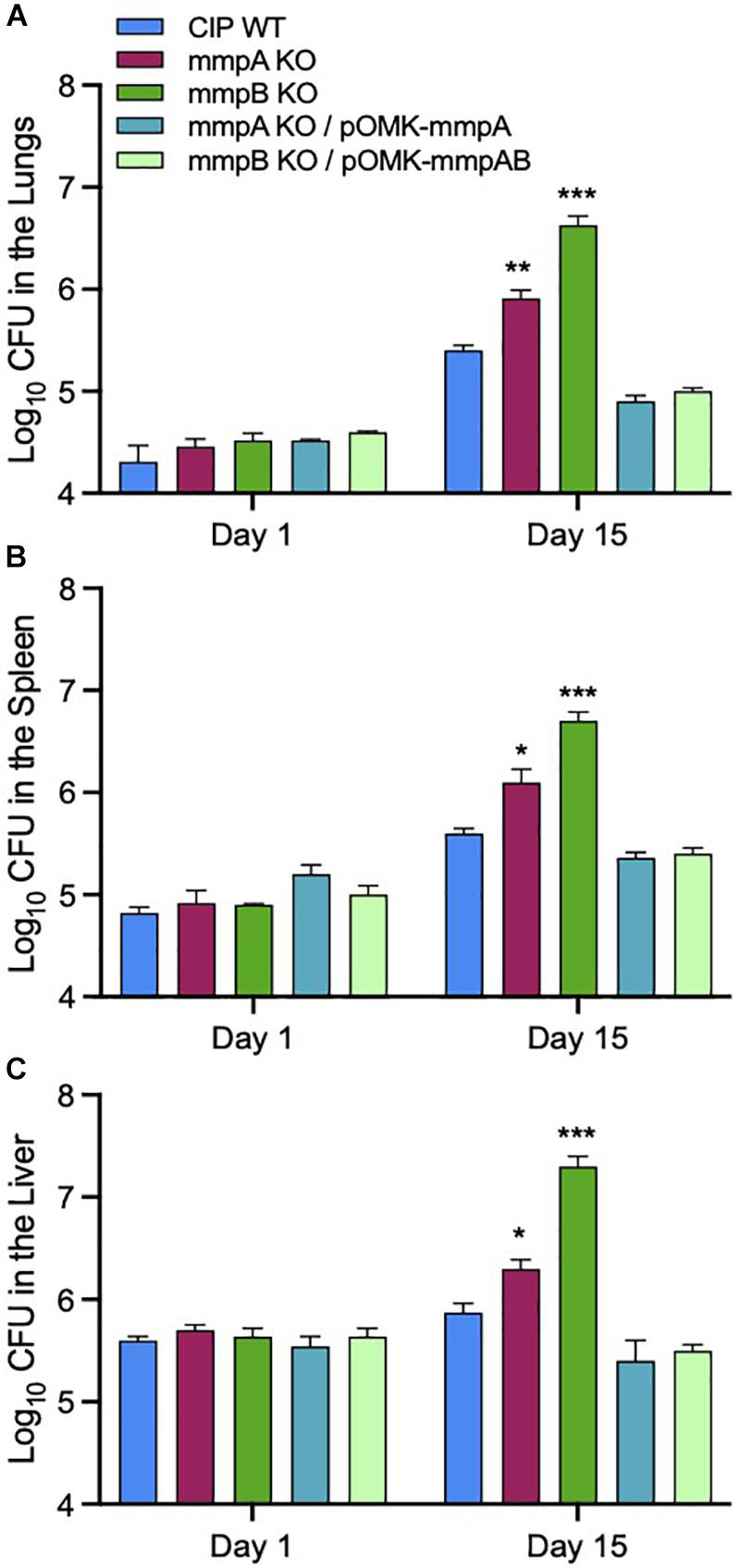
Increased bacterial load in the lungs, spleens and livers of SCID mice infected with the *Mmas* porin knock-out strains. Bacterial counts in the lungs **(A)**, spleens **(B)** and livers **(C)** on days 1 and 15 from SCID mice intravenously infected the WT, porin mutant and complemented mutant strains. Results are expressed as the average (*n* = 5) of the bacterial load in each group expressed as Log10 CFU ± standard error mean (SEM). Growth of the two porin knock-out mutants was significantly higher than that of the WT parent strain pursuant to the Student’s *t*-test (*P < 0.0005; **P < 0.00005; ***P < 0.000005).

We used a SCID mouse intravenous infection model to compare the virulence of *Mmas* WT, mutant and complemented mutant strains *in vivo*. The rationale for using this immunocompromised mouse model is related to the difficulty of establishing a high-level pulmonary infection with rapidly growing mycobacteria in immunocompetent mice (Obregon-Henao et al., 2015). Moreover, this model more closely reflects an infection which would occur in immunocompromised patients, a population particularly at risk for NTM infections (Chan et al., 2010; Prevots and Marras, 2015). Following intravenous infection, the bacterial loads in the lung, spleen and liver were quantified on days 1 and 15 post-infection. Consistent with the observations made in infected BMDM, infection with the *mmpA* and *mmpB* knock-out mutants resulted in increased bacterial loads in the lungs, spleen and liver at 15 days relative to the infection with the WT parent strain, with the *mmpB* mutant displaying the most dramatic increase in virulence (Figure 7). Virulence was restored to or below WT levels upon genetic complementation of the *mmpB* mutant with both porin genes, and that of the *mmpA* mutant with a WT copy of *mmpA*. The *mmpB* mutant complemented with both porin genes was used in this *in vivo* study because of the limited impact of complementing this mutant with *mmpB* only in murine macrophages (i.e., replication rate significantly above that of the WT parent strain) (Figures 6A,B).

**FIGURE 8:**
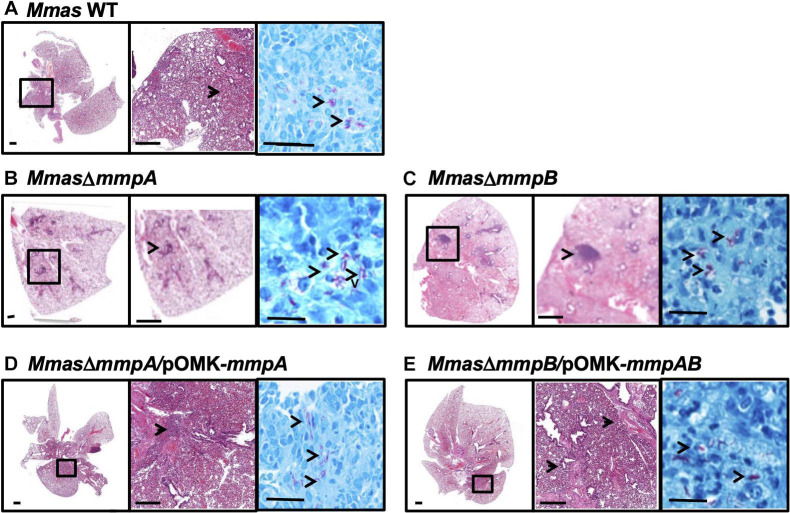
Increased pulmonary pathology in the lungs of SCID mice infected with the *mmpB* knock-out. **(A–E)** Show representative photomicrographs of hematoxylin and eosin- and acid-fast bacilli-stained slides from the lungs of SCID mice intravenously infected with *Mmas* CIP108297 **(A)**, the *mmpA* mutant **(B)**, the *mmpB* mutant **(C)**, the *mmpA* mutant complemented with *mmpA* **(D)** and the *mmpB* mutant complemented with *mmpA* + *mmpB* **(E)**. As early as 15 days after infection, increased granulomatous lesions and thickening of the interstitium indicative of increased inflammation were observed in *mmpB-*infected mice and, to a lesser extent in *mmpA-*infected mice, compared to the other groups. Magnification (– 1x, – 10x and —100x magnification).

Lung histopathology mirrored these observations in that more severe peribronchiolar inflammatory infiltrates and increased cellular foci with early granuloma formation were observed in the lung tissues of the animals infected with the *mmpB* knock-out strain (Figure 8C). In comparison, mice infected with the WT parent and the complemented mutant strains displayed similar lung histopathology with less inflammatory foci and pulmonary involvement, and more healthy lung tissue (Figures 8A,D,E). The *mmpA* mutant displayed an intermediate phenotype (Figure 8B).

## Discussion

The results of our combined genomic and genetic studies indicate that MABSC isolates generally harbor one or two Msp-like porins at the *mmpA/mmpB* locus and that MmpA is the predominant one in terms of level of expression and contribution to nutrient uptake (as measured by [^14^C]-glucose uptake) when both porins are present. The disruption of *mmpA* had a moderate effect on growth in GAS medium and a more marked effect on growth in glucose-based medium. Neither the disruption of *mmpA* or *mmpB* had any significant effect on the antibiotic susceptibility profile of *Mmas* CIP108297. Likewise, neither mutant showed increased resistance to GTA *in vitro*. The latter result and the fact that prototypical GTA-resistant outbreak isolates from Brazil (CRM-019 and CRM-020) have two porins identical in sequence to those of the GTA-susceptible isolates CRM-0270 and CIP108297 (Supplementary Figure 4; Duarte et al., 2009; Davidson et al., 2013) suggest that Msp-type porins have no involvement in the high-level GTA resistance phenotype of the Brazilian outbreak isolates, in contrast to the situation in GTA-resistant *M. smegmatis* and *M. chelonae* laboratory strains and clinical isolates described earlier (Svetlikova et al., 2009).

The significant increase in virulence of the *mmpA* and *mmpB* knock-out mutants in murine macrophages and in SCID mice indicates that the primary impact of alterations in surface porin expression may be on the interactions of MABSC with the host as has been reported in a number of pathogenic bacteria (Achouak et al., 2001; Skurnik et al., 2013; Geisinger and Isberg, 2017; Lee et al., 2017). The fact that the *mmpB* knock-out displays the greatest intracellular replication rate and increase in pathogenicity of the two mutants, yet is the one whose ability to import glucose is the least affected, indicates that this increase in virulence is independent from channel activity and, instead, points to the differential impact of expressing surface MmpB vs. surface MmpA on MABSC’s interactions with the host.

The increased virulence of the *mmpB* and *mmpA* mutants did not correlate with increased uptake by macrophages, cytotoxicity toward infected macrophages or enhanced resistance to nitrosative stress suggestive of the involvement of other virulence mechanisms whose determination will be the object of future studies. Based on what is known of the roles of porins in the virulence of other bacterial pathogens, including CF pathogens, other contributing factors may include decreased susceptibility of the *Mmas* porin mutants to killing by antimicrobial peptides and other host-derived bactericidal compounds, altered interactions with components of the complement cascades, and alterations in their interactions with other cell types than macrophages not analyzed herein (e.g., epithelial cells) (Achouak et al., 2001; Sharbati-Tehrani et al., 2005; Fabrino et al., 2009; Purdy et al., 2009; Skurnik et al., 2013; Lee et al., 2017). Potential changes in the transcription of other, as yet unidentified, genes resulting from the loss of porin expression as reported in carbapenem-resistant *Pseudomonas aeruginosa oprD* mutants (Skurnik et al., 2013), could further contribute to these virulence-associated phenotypic changes.

Consistent with porin content playing a role in the ability of MABSC to evade the host’s defenses, we observed significant DNA rearrangements affecting the *mmpA/mmpB* locus of some of the 1,382 MABSC clinical isolates analyzed in this study. That these DNA rearrangements reflect an attempt of these isolates to overcome antibiotic treatment is unlikely given the lack of significant changes in the antibiotic susceptibility profile of *Mmas* CIP108297 following the disruption of *mmpA* or *mmpB*.

The predominant changes observed longitudinally within CF patients were changes in the number of *mmpA/mmpB* porin genes. However, where short indels were observed longitudinally, they were all found to occur in *mmpA*, which could suggest that the phenotypic effects of the *mmpA* knock-out are specifically under selection in the context of adaption to the CF lung environment. The impact on virulence of fusing the *mmpA* and *mmpB* genes observed in a number of clinical isolates is impossible to predict without dedicated experimentation but is expected to vary with the point of fusion since early fusion points will result in strains expressing mostly an MmpB-type porin and late fusion point will result, on the contrary, in strains expressing mostly a MmpA-like porin. With the data available, we found no evidence to suggest that a particular *mmpA/mmpB* fusion was being selected for, neither within our global collection of MABSC isolates nor in within-patient longitudinal isolates. Finally, we found no evidence that the porin locus was playing a role in the adaptation of clustered MABSC lineages to increased virulence or transmission as we observed no significant association between the number of *mmp*-like porin genes encoded at the *mmpA*/*mmpB* porin locus and whether an isolate had been classified as clustered or unclustered (data not shown) (Bryant et al., 2016). However, 60% of the isolates that encoded only one *mmp*A/*mmpB* porin gene (discounting the isolates from Brazil as these are all associated with a single outbreak), occurred in just three of the clusters defined by Bryant et al. (2016, Figure 2), which suggests that, whilst porin genes may not be playing a role in the increased virulence/transmission of all clustered isolates, they could be contributing to the adaptation of particular lineages.

## Data Availability Statement

The original contributions presented in the study are included in the article/Supplementary Material, further inquiries can be directed to the corresponding author/s.

## Ethics Statement

The animal study was reviewed and approved by Animal Care and Usage Committee of Colorado State University.

## Author Contributions

VM, MJ, DO, RF, JB, and JP conceived, designed experiments, and analyzed the data. VM generated the recombinant strains and characterized *in vitro* phenotypes. IE analyzed the predicted composition of the porin locus from the global population of MABSC isolates. DV, KB, CS, and MS performed the mouse and macrophage infection studies. MJ and DO wrote the manuscript. All authors reviewed and approved the manuscript.

## Conflict of Interest

The authors declare that the research was conducted in the absence of any commercial or financial relationships that could be construed as a potential conflict of interest.
