# Supplementary material for: Increased Virulence of Outer Membrane Porin Mutants of Mycobacterium abscessus
Source: Front Microbiol. 2021 Jul 14;12:706207. doi: 10.3389/fmicb.2021.706207 (PMC8317493; doi:10.3389/fmicb.2021.706207)
Supplement: Supplementary file 1 [file Data_Sheet_1.PDF]

Supplementary Material

Supplementary Table

Table S1: List of primers used in this study

Primer Name	Sequence
**Porin knock-out and pOMK complementation**	
MmpA Fw (*Spe*I)	5'-CATCAT ACTAGT ACTTGCGGACGTCTTGGGTTTGG-3'
MmpA Rv (*Not*I)	5'-CATCAT GCGGCCGC GTAGGCCACGCGGAACCGATCA-3'
MmpB Fw (*Spe*I)	5'-CATCAT ACTAGT GGACACTGCATGTACACGGGATC-3'
MmpB Rv (*Not*I)	5'-CATCAT GCGGCCGC GAGGCAGAGCTGCTCGTGGAGA-3'
MptRv	5'-TGGTCACCAAGTTCAGCGACG-3'
MscLFw	5'-TGGCGATTCAGATCATCGAGC-3'
**qRT-PCR**	
RTMmpA Fw	5'-GGGACTGATAGGAACAGAATGAAG-3'
RTMmpA Rv	5'-ACAAGAGTCATGAGCAGAGC-3'
RTMmpB Fw	5'-TGAGAACGGTTGGCATCC-3'
RTMmpB Rv	5'-GACGACGAGTATCAGTGACG-3'
SigA Fw	5'-CGTTCCTGGACCTGATTCAG-3'
SigA Rv	5'-GTACGTCGAGAACTTGTAACCC-3'

Supplementary Figures

Figure S1: Sequence alignment of the two *Mmas* CIP108297 porins, MmpA and MmpB, with MspA from *M. smegmatis*.

Amino acid sequence alignment of the full-size MmpA and MmpB porins from *Mmas* CIP108297 with MspA from *M. smegmatis*, including signal peptides (underlined).

```
               1                                                 50
     MspA                  MKAISRV LIAMVAAIAA LFTSTGTSHA GLDNELSLVD
     MmpA         MKLLSKV SGWARRGVLA VGALLMTLVA LIATTATAHA GLDDELTLVD
     MmpB      MRTVGIRRVV QSTLTSLILV VGMVGLTVIG ----TGTAHA GLDDELTLVD
Consensus      .........V .......... .......... ....T.T.HA GLD.EL.LVD

               51                                               100
     MspA      GQDRTLTVQQ WDTFLNGVFP LDRNRLTREW FHSGRAKYIV AGPGADEFEG
     MmpA      GKGRLLRIQQ WDTFLNGVFP LDRNRLTREW FHSGRAVYEV TGPGSDAFEG
     MmpB      GKGRLLRIQQ WDTFLNGVFP LDRNRLTREW FHSGRAAYEV TGAGSDTFEG
Consensus      G..R.L..QQ WDTFLNGVFP LDRNRLTREW FHSGRA.Y.V .G.G.D.FEG

               101                                              150
     MspA      TLELGYQIGF PWSLGVGINF SYTTPN--IL IDDGDITAPP FGLNSVITPN
     MmpA      TLELGYQVGY PWSLGVGLNF NYTTPNTSIL YGIPNAFGGS PEASYVQTTN
     MmpB      TLELGYQVGY PWSLGVGLNF NYTTPNTSIL YGIPNAFGGT PEASYVQTTN
Consensus      TLELGYQ.G. PWSLGVG.NF .YTTPN..IL .......... .....V.T.N

               151                                              200
     MspA      LFPGVSISAD LGNGPGIQEV ATFSVDVSGA EGGVAVSNAH GTVTGAAGGV
     MmpA      LLPSAGINVD LGNGPGIQEV ATFSVAVAGP KGAVAVSNAH GTVTGAAGGV
     MmpB      LLPSAGINVD LGNGPGIQEV ATFSVAIAGP KGAVAVSNAH GTVTGAAGGV
Consensus      L.P...I..D LGNGPGIQEV ATFSV...G. .G.VAVSNAH GTVTGAAGGV

               201                       226
     MspA      LLRPFARLIA STGDSVTTYG EPWNMN
     MmpA      LLRPYARLIS SAGDSVTTYG ETWDMK
     MmpB      LLRPYARLIS SAGDSVTTYG ETWDMK
Consensus      LLRP.ARLI. S.GDSVTTYG E.W.M.
```

<u>Figure S2</u>: GTA susceptibility of *Mmas* CIP108297, CRM-019 and the *mmpA* and *mmpB* porin knock-out mutants.

Results are expressed as CFU counts upon exposure of the test organisms to 0.5 or 2.2% GTA (under the formulated form of Cidex®, Johnson & Johnson) for 0, 5 and 30 min. CRM-019 is a GTA-resistant outbreak isolate from Brazil [Duarte *et al.*, 2009; Burgess *et al.*, 2017].

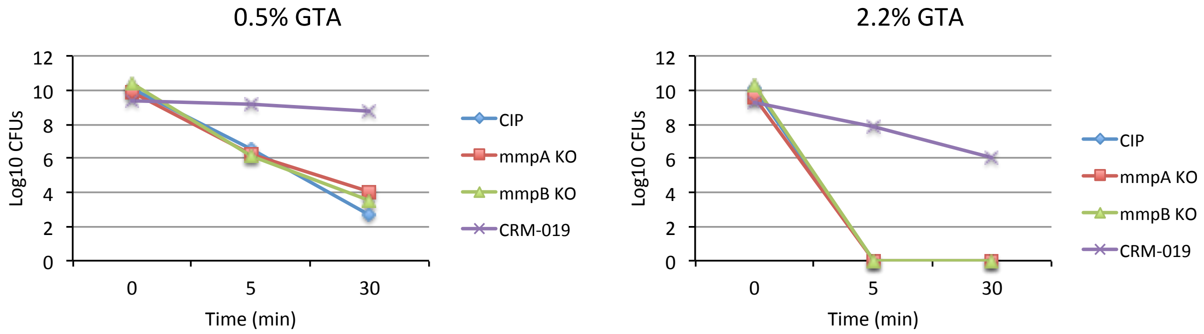

<u>Figure S3</u>: Susceptibility of wild-type *Mmas* and the *mmpA* and *mmpB* porin knock-out mutants to NO *in vitro*.

Shown is the viability of the *Mmas* WT, mutant and complemented mutant strains prior (time 0) and after exposure to the NO donor Spermine NONOate (10 mM) for 4 or 24 hours.

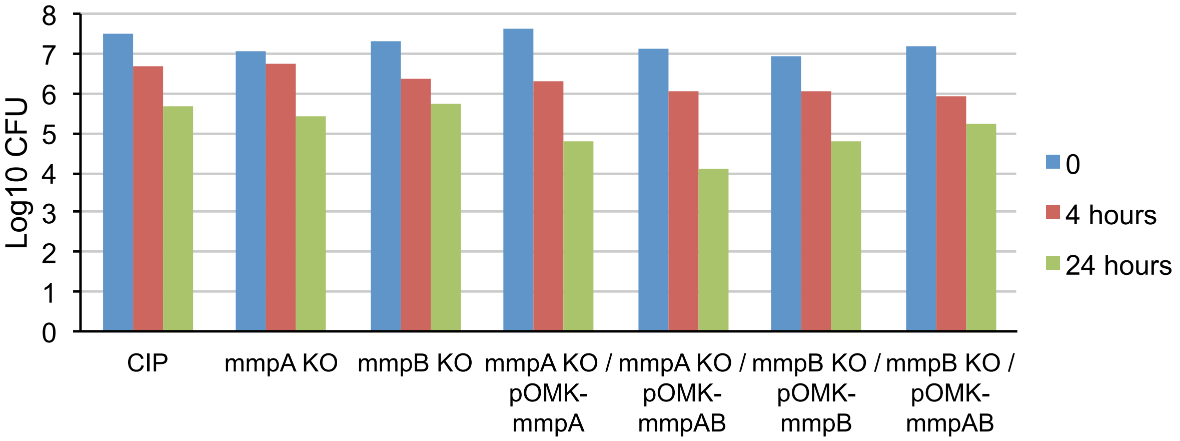

<u>Figure S4</u>: Nucleotide and amino acid sequence alignment of the *mmpA* and *mmpB* region of *Mmas* strains CRM-0020 and CRM-0270 and CIP108297.

CRM-020 is a GTA-resistant outbreak isolate from Brazil whereas CRM-270 is a genetically closely related isolate that is susceptible to GTA [Duarte *et al.*, 2009; Burgess *et al.*, 2017].

<u>MmpA protein sequence alignment</u>

```
           1                                                  50
      CIP  MKLLSKVSGW ARRGVLAVGA LLMTLVALIA TTATAHAGLD DELTLVDGKG
   CRM270  MKLLSKVSGW ARRGVLAVGA LLMTLVALIA TTATAHAGLD DELTLVDGKG
   CRM020  MKLLSKVSGW ARRGVLAVGA LLMTLVALIA TTATAHAGLD DELTLVDGKG
Consensus  MKLLSKVSGW ARRGVLAVGA LLMTLVALIA TTATAHAGLD DELTLVDGKG

           51                                                100
      CIP  RLLRIQQWDT FLNGVFPLDR NRLTREWFHS GRAVYEVTGP GSDAFEGTLE
   CRM270  RLLRIQQWDT FLNGVFPLDR NRLTREWFHS GRAVYEVTGP GSDAFEGTLE
   CRM020  RLLRIQQWDT FLNGVFPLDR NRLTREWFHS GRAVYEVTGP GSDAFEGTLE
Consensus  RLLRIQQWDT FLNGVFPLDR NRLTREWFHS GRAVYEVTGP GSDAFEGTLE

           101                                               150
      CIP  LGYQVGYPWS LGVGLNFNYT TPNTSILYGI PNAFGGSPEA SYVQTTNLLP
   CRM270  LGYQVGYPWS LGVGLNFNYT TPNTSILYGI PNAFGGSPEA SYVQTTNLLP
   CRM020  LGYQVGYPWS LGVGLNFNYT TPNTSILYGI PNAFGGSPEA SYVQTTNLLP
Consensus  LGYQVGYPWS LGVGLNFNYT TPNTSILYGI PNAFGGSPEA SYVQTTNLLP

           151                                               200
      CIP  SAGINVDLGN GPGIQEVATF SVAVAGPKGA VAVSNAHGTV TGAAGGVLLR
   CRM270  SAGINVDLGN GPGIQEVATF SVAVAGPKGA VAVSNAHGTV TGAAGGVLLR
   CRM020  SAGINVDLGN GPGIQEVATF SVAVAGPKGA VAVSNAHGTV TGAAGGVLLR
Consensus  SAGINVDLGN GPGIQEVATF SVAVAGPKGA VAVSNAHGTV TGAAGGVLLR

           201                   223
      CIP  PYARLISSAG DSVTTYGETW DMK
   CRM270  PYARLISSAG DSVTTYGETW DMK
   CRM020  PYARLISSAG DSVTTYGETW DMK
Consensus  PYARLISSAG DSVTTYGETW DMK
```

<u>MmpB protein sequence alignment</u>

```
           1                                                  50
      CIP  MRTVGIRRVV QSTLTSLILV VGMVGLTVIG TGTAHAGLDD ELTLVDGKGR
   CRM270  MRTVGIRRVV QSALTSLILV VGMVGLTVIG TGTAHAGLDD ELTLVDGKGR
   CRM020  MRTVGIRRVV QSALTSLILV VGMVGLTVIG TGTAHAGLDD ELTLVDGKGR
Consensus  MRTVGIRRVV QStLTSLILV VGMVGLTVIG TGTAHAGLDD ELTLVDGKGR

           51                                                100
      CIP  LLRIQQWDTF LNGVFPLDRN RLTREWFHSG RAAYEVTGAG SDTFEGTLEL
   CRM270  LLRIQQWDTF LNGVFPLDRN RLTREWFHSG RAAYEVTGAG SDTFEGTLEL
   CRM020  LLRIQQWDTF LNGVFPLDRN RLTREWFHSG RAAYEVTGAG SDTFEGTLEL
Consensus  LLRIQQWDTF LNGVFPLDRN RLTREWFHSG RAAYEVTGAG SDTFEGTLEL

           101                                               150
      CIP  GYQVGYPWSL GVGLNFNYTT PNTSILYGIP NAFGGTPEAS YVQTTNLLPS
   CRM270  GYQVGYPWSL GVGLNFNYTT PNTSILYGIP NAFGGSPEAS YVQTTNLLPS
   CRM020  GYQVGYPWSL GVGLNFNYTT PNTSILYGIP NAFGGSPEAS YVQTTNLLPS
Consensus  GYQVGYPWSL GVGLNFNYTT PNTSILYGIP NAFGGtPEAS YVQTTNLLPS
```

```
          151                                                200
      CIP AGINVDLGNG PGIQEVATFS VAIAGPKGAV AVSNAHGTVT GAAGGVLLRP
   CRM270 AGINVDLGNG PGIQEVATFS VAIAGPKGAV AVSNAHGTVT GAAGGVLLRP
   CRM020 AGINVDLGNG PGIQEVATFS VAVAGPKGAV AVSNAHGTVT GAAGGVLLRP
Consensus AGINVDLGNG PGIQEVATFS VA!AGPKGAV AVSNAHGTVT GAAGGVLLRP

          201                   222
      CIP YARLISSAGD SVTTYGETWD MK
   CRM270 YARLISSAGD SVTTYGETWD MK
   CRM020 YARLISSAGD SVTTYGETWD MK
Consensus YARLISSAGD SVTTYGETWD MK
```

<u>Porin region DNA sequence alignment</u>

The open reading frames corresponding to *mmpA* and *mmpB* are highlighted in yellow.

```
          1                                                   50
CIP       AATTGGGGGA TGGCTACTTC CGGCTCCAGG CCCGCCTGGG GGCAGGTGAT
CRM270               TGGCTACTTC CGGCTCCAGG CCCGCCTGGG GGCAGGTGAT
CRM020          GGGA TGGCTACTTC CGGCTCCAGG CCCGCCTGGG GGCAGGTGAT
Consensus            TGGCTACTTC CGGCTCCAGG CCCGCCTGGG GGCAGGTGAT

          51                                                 100
CIP       GCGAGGGCTA CCAGATCGAA TGTTCAGATT GCCGCGCCGT CACCGTGCCG
CRM270    GCGAGGGCTA CCAGATCGAA TGTTCAGATT GCCGCGCCGT CACCGTGCCG
CRM020    GCGAGGGCTA CCAGATCGAA TGTTCAGATT GCCGCGCCGT CACCGTGCCG
Consensus GCGAGGGCTA CCAGATCGAA TGTTCAGATT GCCGCGCCGT CACCGTGCCG

          101                                                150
CIP       ATATCGTCCT TCTGTCCGTC GCTAAGGAAA TTCTCATGTT CGCCATGAGG
CRM270    ATATCGTCCT TCTGTCCGTC GCTAAGGAAA TTCTCATGTT CGCCATGAGG
CRM020    ATATCGTCCT TCTGTCCGTC GCTAAGGAAA TTCTCATGTT CGCCATGAGG
Consensus ATATCGTCCT TCTGTCCGTC GCTAAGGAAA TTCTCATGTT CGCCATGAGG

          151                                                200
CIP       GGACTGATAG GAACAGAATG AAGCTGTTGA GCAAGGTATC GGGCTGGGCC
CRM270    GGACTGATAG GAACAGAATG AAGCTGTTGA GCAAGGTATC GGGCTGGGCC
CRM020    GGACTGATAG GAACAGAATG AAGCTGTTGA GCAAGGTATC GGGCTGGGCC
Consensus GGACTGATAG GAACAGAATG AAGCTGTTGA GCAAGGTATC GGGCTGGGCC

          201                                                250
CIP       CGCCGCGGTG TGCTGGCGGT CGGCGCTCTG CTCATGACTC TTGTTGCTCT
CRM270    CGCCGCGGTG TGCTGGCGGT CGGCGCTCTG CTCATGACTC TTGTTGCTCT
CRM020    CGCCGCGGTG TGCTGGCGGT CGGCGCTCTG CTCATGACTC TTGTTGCTCT
Consensus CGCCGCGGTG TGCTGGCGGT CGGCGCTCTG CTCATGACTC TTGTTGCTCT

          251                                                300
CIP       CATTGCGACG ACGGCGACGG CGCATGCGGG TCTGGACGAT GAACTGACGC
CRM270    CATTGCGACG ACGGCGACGG CGCATGCGGG TCTGGACGAT GAACTGACGC
CRM020    CATTGCGACG ACGGCGACGG CGCATGCGGG TCTGGACGAT GAACTGACGC
Consensus CATTGCGACG ACGGCGACGG CGCATGCGGG TCTGGACGAT GAACTGACGC
```

```
           301                                                350
CIP        TGGTTGATGG CAAGGGGCGC TTGCTGCGGA TCCAGCAGTG GGACACCTTT
CRM270     TGGTTGATGG CAAGGGGCGC TTGCTGCGGA TCCAGCAGTG GGACACCTTT
CRM020     TGGTTGATGG CAAGGGGCGC TTGCTGCGGA TCCAGCAGTG GGACACCTTT
Consensus  TGGTTGATGG CAAGGGGCGC TTGCTGCGGA TCCAGCAGTG GGACACCTTT

           351                                                400
CIP        CTCAACGGTG TGTTTCCGTT GGACCGCAAC CGCCTGACGC GTGAGTGGTT
CRM270     CTCAACGGTG TGTTTCCGTT GGACCGCAAC CGCCTGACGC GTGAGTGGTT
CRM020     CTCAACGGTG TGTTTCCGTT GGACCGCAAC CGCCTGACGC GTGAGTGGTT
Consensus  CTCAACGGTG TGTTTCCGTT GGACCGCAAC CGCCTGACGC GTGAGTGGTT

           401                                                450
CIP        CCACTCGGGC CGTGCGGTTT ATGAGGTGAC GGGTCCGGGT TCGGATGCGT
CRM270     CCACTCGGGC CGTGCGGTTT ATGAGGTGAC GGGTCCGGGT TCGGATGCGT
CRM020     CCACTCGGGC CGTGCGGTTT ATGAGGTGAC GGGTCCGGGT TCGGATGCGT
Consensus  CCACTCGGGC CGTGCGGTTT ATGAGGTGAC GGGTCCGGGT TCGGATGCGT

           451                                                500
CIP        TTGAGGGCAC CCTGGAGTTG GGTTACCAGG TTGGTTACCC GTGGTCGTTG
CRM270     TTGAGGGCAC CCTGGAGTTG GGTTACCAGG TTGGTTACCC GTGGTCGTTG
CRM020     TTGAGGGCAC CCTGGAGTTG GGTTACCAGG TTGGTTACCC GTGGTCGTTG
Consensus  TTGAGGGCAC CCTGGAGTTG GGTTACCAGG TTGGTTACCC GTGGTCGTTG

           501                                                550
CIP        GGTGTGGGTT TGAACTTCAA CTACACGACG CCCAACACCT CGATTCTCTA
CRM270     GGTGTGGGTT TGAACTTCAA CTACACGACG CCCAACACCT CGATTCTCTA
CRM020     GGTGTGGGTT TGAACTTCAA CTACACGACG CCCAACACCT CGATTCTCTA
Consensus  GGTGTGGGTT TGAACTTCAA CTACACGACG CCCAACACCT CGATTCTCTA

           551                                                600
CIP        CGGTATTCCG AACGCGTTCG GCGGTAGCCC GGAAGCGTCA TATGTGCAGA
CRM270     CGGTATTCCG AACGCGTTCG GCGGTAGCCC GGAAGCGTCA TATGTGCAGA
CRM020     CGGTATTCCG AACGCGTTCG GCGGTAGCCC GGAAGCGTCA TATGTGCAGA
Consensus  CGGTATTCCG AACGCGTTCG GCGGTAGCCC GGAAGCGTCA TATGTGCAGA

           601                                                650
CIP        CGACCAACCT GTTGCCCAGT GCCGGTATCA ATGTCGACTT GGGCAACGGC
CRM270     CGACCAACCT GTTGCCCAGT GCCGGTATCA ATGTCGACTT GGGCAACGGC
CRM020     CGACCAACCT GTTGCCCAGT GCCGGTATCA ATGTCGACTT GGGCAACGGC
Consensus  CGACCAACCT GTTGCCCAGT GCCGGTATCA ATGTCGACTT GGGCAACGGC

           651                                                700
CIP        CCCGGCATTC AGGAAGTCGC CACCTTCTCG GTAGCGGTTG CAGGTCCGAA
CRM270     CCCGGCATTC AGGAAGTCGC CACCTTCTCG GTAGCGGTTG CCGGTCCGAA
CRM020     CCCGGCATTC AGGAAGTCGC CACCTTCTCG GTAGCGGTTG CCGGTCCGAA
Consensus  CCCGGCATTC AGGAAGTCGC CACCTTCTCG GTAGCGGTTG CaGGTCCGAA

           701                                                750
CIP        GGGTGCGGTG GCGGTGTCCA ACGCGCACGG CACCGTGACC GGTGCAGCCG
CRM270     GGGTGCGGTG GCGGTGTCCA ACGCGCACGG CACCGTGACC GGTGCAGCCG
CRM020     GGGTGCGGTG GCGGTGTCCA ACGCGCACGG CACCGTGACC GGTGCAGCCG
Consensus  GGGTGCGGTG GCGGTGTCCA ACGCGCACGG CACCGTGACC GGTGCAGCCG
```

```
              751                                                 800
     CIP      GTGGTGTGCT GCTTCGTCCC TACGCGCGTT TGATCAGTTC CGCCGGGGAC
  CRM270      GTGGTGTGCT GCTTCGTCCC TACGCGCGTT TGATCAGTTC CGCCGGGGAC
  CRM020      GTGGTGTGCT GCTTCGTCCC TACGCGCGTT TGATCAGTTC CGCCGGGGAC
Consensus     GTGGTGTGCT GCTTCGTCCC TACGCGCGTT TGATCAGTTC CGCCGGGGAC

              801                                                 850
     CIP      AGTGTGACCA CCTACGGCGA GACCTGGGAC ATGAAGTAGG ACGGGGC--A
  CRM270      AGTGTGACCA CCTACGGCGA GACCTGGGAC ATGAAGTAGG ACGGGGC--A
  CRM020      AGTGTGACCA CCTACGGCGA GACCTGGGAC ATGAAGTAGG ACGGGGCGCA
Consensus     AGTGTGACCA CCTACGGCGA GACCTGGGAC ATGAAGTAGG ACGGGGC..A

              851                                                 900
     CIP      CCCCTCCCGC AACCAGGCGC CAAGGCCCAC TCCCATATCG GTCATTCGCG
  CRM270      CCCCTCCCGC AACCAGGCGC CAAGGCCCAC TCCCATATCG GCCATTCGCG
  CRM020      CCCCTCCCGC AACCAGGCGC CAAGGCCCAC TCCCATATCG GCCATTCGCG
Consensus     CCCCTCCCGC AACCAGGCGC CAAGGCCCAC TCCCATATCG GtCATTCGCG

              901                                                 950
     CIP      GACGGTTGCT CACGATGCCT CGTTGCGGTG TTCGCGGCCG TTGGGCCGGT
  CRM270      GACGGTTGCT CACGATGCCT CGTTGCGGTG TTCGCGGCCG TTGGGCCGGT
  CRM020      GACGGTTGCT CACGATGCCT CGTTGCGGTG TTCGCGGCCG TTGGGCCGGT
Consensus     GACGGTTGCT CACGATGCCT CGTTGCGGTG TTCGCGGCCG TTGGGCCGGT

              951                                                1000
     CIP      CTTGGGGCAA CGTCGGGCGC AAGGGTGTTG TCGCGCTGAC ACTGCGCACG
  CRM270      CTTGGGGCAA CGTCGGGCGC AAGGGTGTTG TCGCGCTGAC ACTGCGCACG
  CRM020      CTTGGGGCAA CGTCGGGCGC AAGGGTGTTG TCGCGCTGAC ACTGCGCACG
Consensus     CTTGGGGCAA CGTCGGGCGC AAGGGTGTTG TCGCGCTGAC ACTGCGCACG

              1001                                               1050
     CIP      GCGCCGGGGT TGGGATGTGC CCGTGCCCTC TCTCGCCGCT ACACGTGCTT
  CRM270      GCGCCGGGGT TGGGATGTGC CCGTGCCCTC TCTCGCCGCT ACACGTGCTT
  CRM020      GCGCCGGGGT TGGGATGTGC CCGTGCCCTC TCTCGCCGCT ACACGTGCTT
Consensus     GCGCCGGGGT TGGGATGTGC CCGTGCCCTC TCTCGCCGCT ACACGTGCTT

              1051                                               1100
     CIP      GATCGGTTCC GCGTGGCTAC GCACGGCAAG ACCTGGGATA TGAAGTAGTC
  CRM270      GATCGGTTCC GCGTGGCTAC GCACGGCAAG ACCTGGGATA TGAAGTAGTC
  CRM020      GATCGGTTCC GCGTGGCTAC GCACGGCAAG ACCTGGGATA TGAAGTAGTC
Consensus     GATCGGTTCC GCGTGGCTAC GCACGGCAAG ACCTGGGATA TGAAGTAGTC

              1101                                               1150
     CIP      GCCATTACTG AATAGGTCCC TGGTCGTAGG ACACTGCATG TGCACGGGAT
  CRM270      GCCATTACTG AATAGGTCCC TGGTCGTAGG ACACTGCATG TGCACGGGAT
  CRM020      GCCATTACTG AATAGGTCCC TGGTCGTAGG ACACTGCATG TGCACGGGAT
Consensus     GCCATTACTG AATAGGTCCC TGGTCGTAGG ACACTGCATG TGCACGGGAT

              1151                                               1200
     CIP      CTGTGTATCT GCTGTGAGTT AGCCTCCCTG AAACATTCAA GAAACTCACA
  CRM270      CTGTGTATCT GCTGTGAGTT AGCCTCCCCG AAACATTCAA GAAACTCACG
  CRM020      CTGTGTATCT GCTGTGAGTT AGCCTCCCCG AAACATTCAA GAAACTCACG
Consensus     CTGTGTATCT GCTGTGAGTT AGCCTCCCtG AAACATTCAA GAAACTCACa
```

```
1201                                               1250
       CIP      GAGATGATTT AGCCGTTACG TTTGGCCCGC CTGAGCAACA ATGTGGTCTT
    CRM270      GAGATGATTT AGCCGTTACG TTTGGCCCGC CTGAGCAACA ATGTGGTCTT
    CRM020      GAGATGATTT AGCCGTTACG TTTGGCCCGC CTGAGCAACA ATGTGGTCTT
 Consensus      GAGATGATTT AGCCGTTACG TTTGGCCCGC CTGAGCAACA ATGTGGTCTT

                1251                                               1300
       CIP      GGCGAGGTAG GCATTTGCCC TGGCGTTCAT TCGCCCGAGA ATTGAGGGAA
    CRM270      GGCGAGGTAG GCATTTGCCC TGGCGTTCAT TCGCCCGAGA ATTGAGGGAA
    CRM020      GGCGAGGTAG GCATTTGCCC TGGCGTTCAT TCGCCCGAGA ATTGAGGGAA
 Consensus      GGCGAGGTAG GCATTTGCCC TGGCGTTCAT TCGCCCGAGA ATTGAGGGAA

                1301                                               1350
       CIP      CCAAATGAGA ACGGTTGGCA TCCGGCGGGT TGTGCAGAGC ACCCTCACGT
    CRM270      CCAAATGAGA ACGGTTGGCA TCCGGCGGGT TGTGCAGAGC GCCCTCACGT
    CRM020      CCAAATGAGA ACGGTTGGCA TCCGGCGGGT TGTGCAGAGC GCCCTCACGT
 Consensus      CCAAATGAGA ACGGTTGGCA TCCGGCGGGT TGTGCAGAGC aCCCTCACGT

                1351                                               1400
       CIP      CACTGATACT CGTCGTCGGC ATGGTGGGCC TGACGGTTAT CGGCACCGGT
    CRM270      CACTGATACT CGTCGTCGGC ATGGTGGGCC TGACGGTTAT CGGCACCGGT
    CRM020      CACTGATACT CGTCGTCGGC ATGGTGGGCC TGACGGTTAT CGGCACCGGT
 Consensus      CACTGATACT CGTCGTCGGC ATGGTGGGCC TGACGGTTAT CGGCACCGGT

                1401                                               1450
       CIP      ACGGCGCATG CGGGTCTGGA CGATGAACTG ACGCTGGTTG ATGGCAAGGG
    CRM270      ACGGCGCATG CGGGTCTGGA CGATGAACTG ACGCTGGTTG ATGGCAAGGG
    CRM020      ACGGCGCATG CGGGTCTGGA CGATGAACTG ACGCTGGTTG ATGGCAAGGG
 Consensus      ACGGCGCATG CGGGTCTGGA CGATGAACTG ACGCTGGTTG ATGGCAAGGG

                1451                                               1500
       CIP      GCGCTTGCTG CGGATCCAGC AGTGGGACAC CTTTCTCAAC GGTGTGTTTC
    CRM270      GCGCTTGCTG CGGATCCAGC AGTGGGACAC CTTTCTCAAC GGTGTGTTTC
    CRM020      GCGCTTGCTG CGGATCCAGC AGTGGGACAC CTTTCTCAAC GGTGTGTTTC
 Consensus      GCGCTTGCTG CGGATCCAGC AGTGGGACAC CTTTCTCAAC GGTGTGTTTC

                1501                                               1550
       CIP      CGTTGGACCG CAACCGCCTG ACGCGTGAGT GGTTCCACTC CGGTCGCGCT
    CRM270      CGTTGGACCG CAACCGCCTG ACGCGTGAGT GGTTCCACTC CGGTCGCGCT
    CRM020      CGTTGGACCG CAACCGCCTG ACGCGTGAGT GGTTCCACTC CGGTCGCGCT
 Consensus      CGTTGGACCG CAACCGCCTG ACGCGTGAGT GGTTCCACTC CGGTCGCGCT

                1551                                               1600
       CIP      GCGTACGAAG TGACGGGTGC GGGCTCGGAC ACCTTCGAGG GCACCCTGGA
    CRM270      GCGTACGAAG TGACGGGTGC GGGCTCGGAC ACCTTCGAGG GCACCCTGGA
    CRM020      GCGTACGAAG TGACGGGTGC GGGCTCGGAC ACCTTCGAGG GCACCCTGGA
 Consensus      GCGTACGAAG TGACGGGTGC GGGCTCGGAC ACCTTCGAGG GCACCCTGGA

1601                                               1650
       CIP      GTTGGGTTAC CAGGTTGGTT ACCCGTGGTC GTTGGGTGTG GGTTTGAACT
    CRM270      GTTGGGTTAC CAGGTTGGTT ACCCGTGGTC GTTGGGTGTG GGTTTGAACT
    CRM020      GTTGGGTTAC CAGGTTGGTT ACCCGTGGTC GTTGGGTGTG GGTTTGAACT
 Consensus      GTTGGGTTAC CAGGTTGGTT ACCCGTGGTC GTTGGGTGTG GGTTTGAACT
```

```
          1651                                                1700
CIP       TCAACTACAC GACGCCCAAC ACCTCGATTC TCTACGGTAT TCCGAACGCG
CRM270    TCAACTACAC GACGCCCAAC ACCTCGATTC TCTACGGTAT TCCGAACGCG
CRM020    TCAACTACAC GACGCCCAAC ACCTCGATTC TCTACGGTAT TCCGAACGCG
Consensus TCAACTACAC GACGCCCAAC ACCTCGATTC TCTACGGTAT TCCGAACGCG

          1701                                                1750
CIP       TTCGGCGGTA CTCCCGAGGC CTCGTATGTG CAGACGACCA ACCTGTTGCC
CRM270    TTCGGCGGTA GCCCGGAAGC GTCATATGTG CAGACGACCA ACCTGTTGCC
CRM020    TTCGGCGGTA GCCCGGAAGC GTCATATGTG CAGACGACCA ACCTGTTGCC
Consensus TTCGGCGGTA ctCCcGAgGC cTCgTATGTG CAGACGACCA ACCTGTTGCC

          1751                                                1800
CIP       CAGTGCCGGT ATCAATGTCG ACTTGGGCAA CGGCCCCGGC ATTCAGGAAG
CRM270    CAGTGCCGGT ATCAATGTCG ACTTGGGCAA CGGCCCCGGC ATTCAGGAAG
CRM020    CAGTGCCGGT ATCAATGTCG ACTTGGGCAA CGGCCCCGGC ATTCAGGAAG
Consensus CAGTGCCGGT ATCAATGTCG ACTTGGGCAA CGGCCCCGGC ATTCAGGAAG

          1801                                                1850
CIP       TCGCCACCTT CTCAGTGGCT ATCGCCGGTC CGAAGGGTGC GGTGGCGGTG
CRM270    TCGCCACCTT CTCAGTGGCT ATCGCCGGTC CGAAGGGTGC GGTGGCGGTG
CRM020    TCGCCACCTT CTCGGTAGCG GTTGCCGGTC CGAAGGGTGC GGTGGCGGTG
Consensus TCGCCACCTT CTCaGTgGCt aTcGCCGGTC CGAAGGGTGC GGTGGCGGTG

          1851                                                1900
CIP       TCCAACGCGC ACGGCACCGT GACCGGTGCA GCCGGTGGTG TGCTGCTTCG
CRM270    TCCAACGCGC ACGGCACCGT GACCGGTGCA GCCGGTGGTG TGCTGCTTCG
CRM020    TCCAACGCGC ACGGCACCGT GACCGGTGCA GCCGGTGGTG TGCTGCTTCG
Consensus TCCAACGCGC ACGGCACCGT GACCGGTGCA GCCGGTGGTG TGCTGCTTCG

          1901                                                1950
CIP       TCCCTACGCG CGTTTGATCA GTTCCGCCGG GGACAGTGTG ACCACCTACG
CRM270    TCCCTACGCG CGTTTGATCA GTTCCGCCGG GGACAGTGTG ACCACCTACG
CRM020    TCCCTACGCG CGTTTGATCA GTTCCGCCGG GGACAGTGTG ACCACCTACG
Consensus TCCCTACGCG CGTTTGATCA GTTCCGCCGG GGACAGTGTG ACCACCTACG

          1951                                    1990
CIP       GCGAGACCTG GGACATGAAG TAACCACCGC CGGACTGG
CRM270    GCGAGACCTG GGACATGAAG TAACT---GC CGGACTGG
CRM020    GCGAGACCTG GGACATGAAG TAACT---GC CGGACTGG
Consensus GCGAGACCTG GGACATGAAG TAACcaccGC CGGACTGG
```

References

Burgess, W., Margolis, A., Gibbs, S., Duarte, R. S., and Jackson, M. (2017) Disinfectant Susceptibility Profiling of Glutaraldehyde-Resistant Nontuberculous Mycobacteria. Infect Control Hosp Epidemiol 38, 784-791

Duarte, R. S., Lourenco, M. C. S., de Souza Fonseca, L. S., Leao, S. C., de Lourdes T., Amorim, E., et al. (2009) An epidemic of postsurgical infections caused by *Mycobacterium massiliense*. *J. Clin. Microbiol.* **47**, 2149-2155
